# Design and application of emotion-oriented play-learning tools for children with autism: a cross-cultural qualitative study of parents and teachers in China and Malaysia

**DOI:** 10.3389/fpsyg.2026.1736058

**Published:** 2026-04-30

**Authors:** Yiming Xing, Nazean Jomhari, Norhanim Zakaria, Xizhen Zhou, Shunfa Lai, Yueting Liu

**Affiliations:** 1 Institute for Advanced Studies, Universiti Malaya, Federal Territory of Kuala Lumpur, Malaysia; 2 Faculty of Computer Science and Information Technology, Universiti Malaya, Federal Territory of Kuala Lumpur, Malaysia; 3 Faculty of Built Environment, Universiti Malaya, Federal Territory of Kuala Lumpur, Malaysia; 4 Office of Student Affairs, Southwest Jiaotong University Hope College, Chengdu, China

**Keywords:** autism spectrum disorder (ASD), cross-cultural comparison, emotion-oriented play-learning tools, home–school collaboration, reflexive thematic analysis, sensory feedback, user-centered design (UCD)

## Abstract

**Background:**

Autistic children experience enduring difficulties with emotion regulation and daily participation. Emotion-oriented play-learning tools (EPLTs, sensory play-learning tools for supporting emotion and self-regulation) are recognized as low-threshold supports across home–school–community settings; however, robust real-world evidence (RWE) on their use, design requirements, and cross-cultural variation remains limited.

**Purpose:**

The purpose of this study is to examine the meanings, applications, and design requirements of emotion-oriented play-learning tools in China and Malaysia, and to identify system-level enablers and barriers to their development and use from stakeholder perspectives.

**Methods:**

Adopting reflexive thematic analysis, we conducted semi-structured interviews with 30 stakeholders (15 in China; 15 in Malaysia)—parents/primary caregivers and frontline teachers. Data were analyzed inductively in NVivo using three-level coding, and the reporting followed the Standards for Reporting Qualitative Research (SRQR).

**Results:**

Five overarching themes were identified: (1) driving factors (heightened emotional difficulties and trend-driven impetus); (2) attitudes to advanced technologies [virtual/augmented reality (VR/AR) and artificial intelligence (AI)]; (3) potentials and applications (multimodal sensory feedback, support for emotional expression, and transition support); (4) design and market requirements (safety, affordability, maintainability, collaboration, and data minimization); and (5) socio-environmental issues (stigma and policy accessibility). Coding frequencies indicated Theme 4 was the most salient, with stronger emphasis among parents than teachers. Attitudes to technology diverged by country—Malaysian participants were generally more open, whereas Chinese participants were more cautious—while socio-environmental constraints were common to both. Overall, Chinese participants offered denser, institution-focused accounts, whereas Malaysian participants emphasized family- and community-based practical coping.

**Conclusions:**

Emotion-oriented play-learning tools may operate as low-threshold, context-adaptable supplement tools in real-world settings. Scalable adoption depends on evidence-informed design; integrated affordability across price, maintenance, and training; and governance frameworks for privacy and explainability. We propose a design paradigm centered on safety, affordability, evidence, and collaboration. This paradigm is intended to guide small-scale RWE pilots, enable home-school data sharing and cross-cultural adaptation, and ultimately enhance quality of life for autistic children—aligning with the Sustainable Development Goals (SDGs) on Good Health and wellbeing, Quality Education, and Reduced Inequality.

## 1 Introduction

Autism spectrum disorder (ASD) is a neurodevelopmental condition characterized by persistent difficulties in social communication and interaction, restricted interests and repetitive behaviors, and frequently accompanied by atypical sensory processing (First et al., 2022). Global prevalence estimates vary across studies and methodologies; according to the World Health Organization (WHO) factsheet, approximately one in 127 children were diagnosed as of 2021 (WHO, 2025). Beyond the diagnostic features, many autistic children experience pronounced emotion dysregulation, heightened stress sensitivity, and challenges in daily functioning, which in turn increase family caregiving burdens and diminish overall quality of life (Kim et al., 2024; Restoy et al., 2024; van Niekerk et al., 2023). At the sensory level, systematic reviews consistently report higher rates of hypo-reactivity and sensory-seeking behaviors relative to typically developing peers, with these sensory differences closely linked to emotion regulation and social participation (Camino-Alarcón et al., 2024; Chen et al., 2024; Patil and Kaple, 2023). Existing research has confirmed that sensory processing disorder in children with autism does not exist in isolation; instead, it is bidirectionally linked to deficits in emotional regulation. Sensory input that is either overloaded or insufficient can directly trigger emotional outbursts (such as screaming or self-injury), while emotional dysregulation further exacerbates sensory sensitivity, creating a maladaptive cycle of “sensory disorder—emotional outbursts—impaired functioning” (Camino-Alarcón et al., 2024).

It is clear that emotional regulation is of vital importance for children with autism. Nowadays, in terms of emotional regulation strategies, multimodal sensory feedback has proven valuable for temporarily stabilizing situations during rapidly escalating emotional episodes such as sudden meltdowns in children (Ning, 2025; Yang and Zhang, 2025). However, a common practical misconception lies in equating “calming” with “effectiveness”: if a child, after being calmed emotionally, does not transition into routine contextual tasks such as “entering the classroom” or “completing assignments,” the intervention remains at the level of immediate relief rather than functional improvement (Schaaf et al., 2018). In the absence of effective preventive measures, “post-outburst intervention” remains the mainstream approach (Micheel et al., 2025). Parents and teachers largely rely on repeated trial and error to determine the intensity threshold and timing of intervention use (Grant et al., 2016). This decision-making process, heavily dependent on personal experience, not only increases time and economic costs (Yaacob et al., 2021) but also, due to the individual differences among children with autism, is prone to the phenomenon of “experience transfer” during decision-making. For example, when institutional teachers use hugging as a soothing technique for different children, it may actually increase the child's stress if the emotional or behavioral causes differ (Than et al., 2024). This often obscures the true benefits of the intervention.

Meanwhile, from a child development perspective, play serves as a vital vehicle for cognitive, emotional, and social growth, while play-learning tools function as mediators that facilitate spontaneous exploration, imitation, and social engagement (Alotaibi, 2024). For autistic children, opportunities for sensory–emotional regulation within safe, familiar, and predictable settings are particularly critical: with the help of appropriate play-learning tools and environmental cues, children with autism can more smoothly transition from states of overexcitement, anxiety, or depression back to a calm and focused state (Schaaf et al., 2024).

In both academic and clinical practice, there are different terms for “sensory-based devices/toys that assist children with autism in emotional regulation,” which can be broadly divided into two categories. One category refers to “rehabilitation therapy devices that assist children with autism in emotional regulation,” which are primarily used in professional therapeutic settings. These are often medical-grade devices (such as professional sensory training instruments) that require operation under the guidance of trained professionals, are relatively costly, and are limited in terms of usage scenarios (Barton et al., 2021; Saleh et al., 2014). The other category refers to “emotion-oriented play-learning tools that assist children with autism in emotional regulation.” These are designed for everyday life contexts, use “play” as the medium, integrate multimodal sensory feedback, and can be used independently by children with the assistance of parents or teachers. They are characterized by convenience and accessibility (Elbeltagi et al., 2023; Peña et al., 2021). The latter, with the core goal of assisting emotional regulation and self-soothing, integrates multimodal sensory feedback such as tactile input, sound and light, and vibration. It can be conveniently used in everyday settings such as families, institutions, and communities. Its core characteristics are “non-therapeutic attributes, suitability for everyday contexts, and providing emotional support through sensory feedback.” Examples include sensory-based tools/toys designed to assist children with autism in emotional regulation and self-soothing, which integrate multimodal sensory feedback such as tactile input, sound and light, and vibration, and can be easily used in daily environments like families, institutions, and communities. These include, but are not limited to, tactile mats, soft comfort toys, sound and light modulation devices, and squeezable stress-relief toys. Their defining features are “non-therapeutic attributes, adaptability to everyday scenarios, and the use of sensory feedback to achieve emotional regulation.”

This study focuses on the second category, “emotion-oriented play-learning tools that assist children with autism in emotional regulation.” To clarify the research boundaries and ensure terminological consistency throughout the text, and in alignment with the core goal of “assisting emotional regulation and adapting to everyday contexts,” we explicitly define these as “Emotion-oriented play-learning tools (EPLTs).” This term is highly consistent with the research context of “sensory-based play-learning tools that assist children with autism in emotional regulation.” It emphasizes the clear distinction between these tools and rehabilitation therapy devices designed for the same purpose—the latter are focused on therapeutic disease rehabilitation and have high operational thresholds. In contrast, EPLTs focus on providing supportive emotional regulation, are easy to use, suitable for multiple contexts, and leverage “play” to reduce children's resistance, making them more readily accepted by families and institutions.

It is important to note that while interventions based on sensory integration theory have shown some positive effects in recent years (Acuña et al., 2025), however, the evidence for single-modality sensory intervention strategies remains limited and their effects are unstable (Wong, 2023). For instance, a randomized controlled trial investigating the use of weighted blankets to improve sleep did not find significant improvements in objective sleep metrics. This finding suggests that we need to exercise caution when promoting EPLTs, ensuring their use is grounded in actual evidence of effectiveness (Cifre et al., 2024).

In existing research, investigations related to the core subject of this study have primarily focused on single-item sensory play-learning tools (e.g., tactile balls; Gee et al., 2020; Hong, 2018). However, research exploring the authentic views and perceptions of parents and teachers of children with autism regarding EPLTs remains limited, and has often overlooked the nuanced design requirements within emotional regulation pathways as well as differences in cultural contexts (López-Nieto et al., 2022; Nuske et al., 2023). Furthermore, variations across different countries and regions in terms of educational systems, family resources, cultural expectations, and the friendliness of public spaces may profoundly influence the accessibility, acceptability, and actual usage patterns of EPLTs (Keating et al., 2024; Lee et al., 2025). However, research adopting a cross-cultural perspective on this topic is currently still scarce.

Based on the identified gaps, this study focuses on the meaning, design features, and usage preferences of EPLTs in authentic contexts. Adopting a cross-cultural perspective and incorporating the perspectives of parents and teachers in China and Malaysia, this research aims to provide an evidence base for the design and interdisciplinary application of genuinely effective EPLTs that are centered on children, caregivers, and special education teachers. Furthermore, grounded in authentic data feedback, it seeks to contribute to improving the quality of life for children with autism, thereby responding to the Sustainable Development Goals (SDG; Nedungadi et al., 2023; WHO, 2025).

## 2 Methods

### 2.1 Study design

This study was designed and reported in accordance with the Standards for Reporting Qualitative Research (SRQR; Dossett et al., 2021), with the full checklist provided in Appendix S1. Adopting a constructivist orientation, we employed reflexive thematic analysis (Braun and Clarke, 2012). An audit trail was maintained throughout the research process, documenting raw data management, iterative codebook revisions, records of discrepancy negotiation, and reflexive memo writing. The following sections describe the sampling strategy, contextual information, data collection and handling procedures, and the techniques used to enhance trustworthiness—including dual independent coding, peer debriefing, and negative case analysis. The study received ethical approval from the University of Malaya Research Ethics Committee (UM.TNC2/UMREC_4398).

### 2.2 Participants and recruitment

We used maximum-variation sampling to recruit two stakeholder groups: (a) parents/primary caregivers and (b) education practitioners, defined as frontline teachers in agencies supporting autistic children. Eligibility was not restricted by age, educational background, or gender, provided participants could communicate adequately. Inclusion criteria are summarized in Supplementary material. For the parent group, participants were required to be first-degree relatives of a child with a confirmed autism spectrum disorder (ASD) diagnosis and to have such a child currently in the family. For the teacher group, participants were frontline staff in relevant institutions—such as rehabilitation, supportive therapy, or early intervention centers— who worked directly with autistic children and held positions likely to have a positive developmental impact. The interview stage participant information is shown in Table 1.

**Table 1:** Participant information description.

Participant ID	Country	Role	Gender	Child's age/ASD characteristics	Teaching experience	Interview method
P01	C	P	F	4y; ASD level1	—	OI
P02	C	P	F	5y; ASD level1	—	OI
P03	C	P	F	6y; ASD level1	—	OI
P04	C	P	F	7y; ASD level1	—	OI
P05	C	P	F	8y; ASD level1	—	OI
P06	C	P	F	7y; ASD level1	—	OI
P07	C	P	F	3y; ASD level1	—	OI
P08	C	P	F	6y; ASD level1	—	OI
P09	C	P	F	3y; ASD level1	—	OI
P10	C	P	F	7y; ASD level1	—	OI
T01	C	T	F	—	2y	OI
T02	C	T	F	—	3y	OI
T03	C	T	F	—	3y	OI
T04	C	T	F	—	5y	OI
T05	C	T	F	—	3y	OI
P01	M	P	F	11y; ASD level1	—	OI
P02	M	P	F	12y; ASD level1	—	PI
P03	M	P	M	9y; ASD level1	—	OI
P04	M	P	F	7y; ASD level1	—	OI
P05	M	P	F	7y; ASD level1	—	PI
P06	M	P	F	13y; ASD level1	—	OI
P07	M	P	F	8y; ASD level1	—	PI
P08	M	P	M	17y; ASD level1	—	OI
P09	M	P	F	21y; ASD level1	—	OI
P10	M	P	F	15y; ASD level1	—	PI
T01	M	T	F	—	3y	OI
T02	M	T	F	—	5y	PI
T03	M	T	M	—	8y	PI
T04	M	T	F	—	4y	OI
T05	M	T	F	—	4y	PI

C, China; M, Malaysia; P, Parent; T, Teacher; —, Not applicable; F, Female; M, Male; OI, Online Interview; PI, Physical Interview.

The Participant ID in this table is the participant code during the interview stage.

The interviews were designed to elicit parents' and teachers' perspectives on EPLTs; all accounts of product use therefore represented proxy reports from these stakeholders. Prior research indicates that parent-proxy reports provide consistent and reliable observational information about children's behaviors (Eiser and Morse, 2001). Given the vulnerability of the study population and the practical challenges of naturalistic data collection, parents of individuals currently over 18 years of age were included, provided their reflections referred to experiences when the child was under 18—a procedure considered both relevant and ethically acceptable (von Wirth et al., 2021).

To ensure that respondents clearly understood the research subject, the core object of this study will be explicitly defined to all participants: EPLTs designed primarily to assist children with autism in emotional regulation and self-soothing. These tools integrate multimodal sensory feedback—such as tactile input, sound and light—and can be conveniently used in settings such as families and institutions. Examples include, but are not limited to, tactile mats, soft comfort toys, and sound and light modulation devices. They are distinct from purely rehabilitation therapy devices (e.g., professional therapeutic instruments) and emphasize emotional support through the medium of “play”.

### 2.3 Data collection

Guided by an expert-reviewed, semi-structured interview protocol, we conducted interviews exploring parents' and teachers' perspectives on the development and use of EPLTs for autistic children. Interviews were carried out either via videoconferencing platforms (e.g., Google Meet, Tencent Meeting) or face-to-face by the researchers, with each session lasting approximately 30 min, reflecting a mixed online–offline approach. With written and verbal consent, all interviews were audio-recorded and subsequently transcribed verbatim.

### 2.4 Data analysis

All interview audio files were transcribed and re-listened to in full by co-authors for verification. Transcripts were subsequently de-identified through the removal of names, precise locations, and institutional identifiers, and each participant was assigned a unique code (P1–P30) with country and role tags. A case codebook was maintained in NVivo 14, documenting the rationale and date for each merge or split decision and preserving version history. Matrix queries were used to generate comparative counts across country × role × theme, and exportable frequency tables were produced to support the visualizations presented in Section 3.8. These comparative counts were used not as endpoints, but as an analytical tool to identify patterns and disparities for deeper qualitative interrogation during the theme review phase.

We followed the six-phase procedure of thematic analysis outlined by (Braun and Clarke 2012): Familiarization → initial coding → theme generation → theme review → theme naming → report production. Inductive, semantic-level coding was undertaken through three iterative cycles, using NVivo 14 as the qualitative analysis tool. To enhance credibility, the interview guide underwent expert review and discussion prior to data collection, and revisions were made until consensus was achieved.

### 2.5 Methodological rigor

We enhanced study quality by following (Lincoln and Guba 1985) four criteria—credibility, dependability, confirmability, and transferability—through standardized interviewer training; dual independent coding with discrepancy negotiation; reflexive memoing of assumptions and decision-making; and the inclusion of representative quotations to support key conclusions. Reporting was cross-checked against the SRQR checklist (Dossett et al., 2021). For procedural consistency, all interviews were conducted by a single researcher (the first author), a PhD candidate in human–computer interaction (HCI) with sustained involvement in school–family collaboration projects related to autism spectrum disorder (ASD). Prior to coding, this researcher documented expectations regarding the perceived “effectiveness of EPLTs” and, during team meetings, routinely examined potential bias signals. The researcher had no hierarchical or collegial relationship with participants, thereby reducing power differentials and role-related bias.

Before coding commenced, the research team completed reflexive memos outlining *a priori* assumptions concerning “the effectiveness of EPLTs,” “the advantages and disadvantages of technological involvement,” and “China–Malaysia differences.” During each round of code merging and theme naming, these memos were revisited to identify potential sources of bias and corresponding mitigation strategies (e.g., negative case analysis to refine theme boundaries). The first author (male; PhD candidate in HCI) had prior experience with ASD-related projects, which could predispose optimism regarding technological feasibility while also enhancing sensitivity to contextualized design issues. To address this, peer debriefing sessions were facilitated by a non-technical researcher to provide critical reflection and balance.

### 2.6 Ethics

This study adhered to the ethical principles outlined in the World Medical Association's *Declaration of Helsinki* for research involving human participants (World Medical Association, 2013). Ethical approval was obtained from the University of Malaya Research Ethics Committee (UM.TNC2/UMREC_4398). To minimize privacy risks, all audio files were securely stored and transcribed exclusively within Malaysia. Prior to analysis, identifiable information was removed, and only de-identified text was used for scholarly collaboration and data interpretation. Before obtaining written and verbal consent, the researchers introduced themselves, explained the study purpose, and described the data collection procedures. Participants who agreed to take part and to audio recording signed written informed consent forms. Interview times and locations were arranged according to participant preference. Participants were informed of their right to withdraw at any time before or during participation. Data protection measures were communicated at each stage, and participants were encouraged to contact the research team with any questions or concerns.

## 3 Results

### 3.1 Participant overview

A total of 30 participants took part in this study, comprising parents/primary caregivers and frontline teachers from both China and Malaysia, with differing proportions across the two stakeholder groups. Their collective experiences encompassed caregiving and educational support for autistic children, spanning the preschool to adolescent stages. Thematic saturation was reached after the 30 interview completed.

### 3.2 Thematic structure

The study explored how parents of autistic children and frontline institutional teachers understood, experienced, and perceived EPLTs.

Analysis of the qualitative dataset identified five overarching themes:

(1) Socio-environmental issues affecting autistic children;
(2) Potential and applications of EPLTs;
(3) Driving factors influencing their development;
(4) Design requirements for EPLTs;
(5) Attitudes toward the integration of advanced technologies.

These major themes were further elaborated into 13 subthemes (see Table 2 below).

**Table 2:** Thematic framework of NVivo qualitative interviews.

Themes	Sub-theme
Socio-environmental issues of autistic children	•Negative social phenomena •Shortcomings of the official government
Potential and application of EPLTs for autistic children	•Sensory feedback is an effective sedative strategy. •Potential of incorporating data recording and analysis functions •Importance of Emotional Toys
Factors driving the development of EPLTs for autistic children	•Emotional problems are becoming increasingly serious •Prospective trends support
Design requirements for EPLTs for autistic children	•Key purchase decision •Requirements for toy detail design •Defects in market exposure •Pricing Strategy
Attitude toward incorporating advanced technological design elements into EPLTs	•Positive attitude •Negative attitude

### 3.3 Socio-environmental issues surrounding autistic children

Interview respondent coding analysis results indicate that both parents and teachers in China and Malaysia have expressed multiple perspectives on the current social environmental issues faced by children with autism. There is a widespread perception that inefficient government support and social inequities form a negative, self-perpetuating cycle. This macro-level challenge is not an isolated phenomenon; rather, it directly impacts the affordability, usage contexts, and practical effectiveness of EPLTs, thus emerging as a significant external factor constraining their broader adoption and application.

#### 3.3.1 Negative social phenomena

Participants widely expressed that the social situation of children with autism urgently needs improvement, noting that discrimination and social exclusion against these children exist in both Chinese and Malaysian social environments. This reality directly drives families' demand for EPLTs—due to low social acceptance, children with autism are prone to emotional outbursts in public settings triggered by external scrutiny and environmental stimuli. Families are thus compelled to rely on portable, discreet EPLTs (such as small soft comfort toys or squeezable stress-relief props) to help their children quickly regulate emotions in public contexts, avoid conflicts, and mitigate the negative impact of social exclusion. Meanwhile, the resources provided by public special education schools—including EPLTs, teaching staff, course fees, and institutional accessibility—fall short of meeting the growing demands arising from the increasing population of children with autism. This forces families to bear the costs of purchasing play-learning tools themselves. The emotional and behavioral challenges associated with autism already impose significant financial strain on families, and the lack of social support resources further restricts their options for acquiring high-quality EPLTs. Some families, facing economic pressures, are even compelled to forgo purchasing such tools altogether, resorting instead to alternative approaches with limited effectiveness. Representative interview excerpts are presented in Table 3.

**Table 3:** Negative social phenomena.

Illustrative quotes	Participant ID
*I just want to say that I hope society pays more attention to autistic children*.	P27
*In public places, neurotypical kids might discriminate against autistic children*.	P28
*No one wants to be friends with my son*.	P8

#### 3.3.2 Deficiencies in governmental governance and support

Parents and teachers of children with autism in both China and Malaysia have put forward suggestions for improving government support, focusing primarily on areas such as compassionate care, financial assistance, public awareness initiatives, and administrative efficiency. The lack of government support directly affects both the development of EPLTs and the purchasing power of families. Specifically, the absence of government subsidies for families means that limited financial resources must be prioritized for professional rehabilitation services for children with autism, leaving families unable to afford high-quality EPLTs. Furthermore, the government has not organized professional training for parents and teachers on the use of EPLTs and complementary therapeutic approaches. Consequently, even when families manage to acquire such tools, they often lack the knowledge to use them effectively, diminishing their potential for emotional support and, in some cases, even triggering resistance from children due to improper use. Additionally, the government has not established unified design and quality standards for EPLTs, resulting in uneven product quality in the market. Some products blur the distinction between EPLTs and rehabilitation, creating mismatches in function and application context, which further undermines family trust and willingness to use these tools. Respondents expressed hope that the government would improve relevant support policies—by providing financial subsidies to reduce the cost of acquiring play-learning tools, offering professional training, and establishing unified standards—to promote the broader adoption of EPLTs and alleviate the caregiving burden on families. Representative interview excerpts are presented in Table 4.

**Table 4:** Recommendations for government improvement.

Illustrative quotes	Participant ID
*I hope government/social institutions can support in these areas: Training for parents and teachers to use the toys R&D grants for local toy developers Evaluation systems so we know whether a toy really works or not*.	P12
*Sometimes the government provides equipment that we don't even know how to use, and we ourselves feel it's not effective*.	P15
*I hope the government will provide more feedback in any form*.	P6

### 3.4 Potential and applications of EPLTs for autistic children

Analysis of participant coding revealed that both parents and teachers articulated diverse perspectives on the developmental potential, expected functions, and perceived importance of EPLTs for autistic children. Participants emphasized that integrating sensory feedback with data recording and visual analytics could enhance users' understanding of these tools and strengthen perceptions of their effectiveness in real-world contexts.

#### 3.4.1 Sensory feedback as a complementary intervention strategy

Drawing on both direct and indirect interview accounts, participants generally agreed that sensory stimulation—both physical and affective—serves as an effective complementary strategy for supporting autistic children. Respondents reported that sensory-based stimuli help regulate emotional states, maintain engagement, and facilitate smoother behavioral transitions.

Observations from institutions providing therapeutic support further corroborated this view: play-learning tools used in practice frequently incorporate multisensory feedback, reinforcing their perceived therapeutic value. Representative examples are summarized in Table 5.

**Table 5:** Sensory feedback as an intervention strategy.

Illustrative quotes	Participant ID
*At home we have a toy room with lots of toys and even a small playground. At school, my son often goes into their sensory room and the teachers say she enjoys it. So yes, some sensory toys do have positive effects*.	P1
*At the therapy center, they use tactile mats with him because he has reduced sensitivity to pain, so they provide tactile stimulation exercises*.	P25
*Generally, there are more sound and light toys because kids are more interested*.	P20

#### 3.4.2 Expectations regarding behavioral data recording and visualization

Most participants believed that incorporating data-recording and visualization functions into play-learning tools could help parents and teachers better understand children's real-time emotional and behavioral states. By analyzing the patterns, triggers, and contextual factors associated with emotional behaviors among autistic children, these tools were regarded as valuable for monitoring and optimizing daily support. Representative quotations are presented in Table 6.

**Table 6:** Expectations regarding behavioral data-recording and analysis.

Illustrative quotes	Participant ID
*Yes, because the information from the tools or application is very valuable for us as parents and therapists to evaluate how the child behavior. It can help improve learning, therapy, and communication management*.	P3
*Once the data is collected, it will be very helpful for AI to provide recommendations to parents or teachers*.	P13
*Recording emotional data and generating reports could help identify patterns and triggers, track progress over time, and allow parents and therapists to tailorinterventions more effectively to the child's specific needs*.	P14

Participants further suggested that these functions could facilitate the evaluation of intervention effectiveness and provide feedback for timely adjustments to support strategies based on objective behavioral data.

However, a small number of respondents expressed concerns about data privacy and product affordability, particularly the higher costs typically associated with devices equipped with data-recording or analytic capabilities.

#### 3.4.3 Perceptions of the importance of EPLTs

Participants demonstrated varied yet consistent recognition of the importance of EPLTs for autistic children. According to their accounts, emotional and behavioral reactions—such as sudden crying, screaming, and self-injurious behavior—were identified as some of the most salient and disruptive factors influencing both children's development and the daily lives of their families.

Within this context, both parents and teachers observed that small or handheld play-based tools with emotional features could help children express and release emotional stress in daily situations. These tools were described as practical and readily accessible tools that supported emotional self-regulation. Representative quotations are presented in Table 7.

**Table 7:** Perceptions of the importance of EPLTs.

Illustrative quotes	Participant ID
*Toys helped him express emotions in a simple way*.	P9
*Soft toys can help reduce stress and provide comfort*.	P14
*Emotional regulation toy stabilize his emotions, yes, and then perhaps it might give him some sense of security in a strange environment*.	P24

### 3.5 Factors driving the development of EPLTs for autistic children

This section interprets the coding results reflecting participants' perspectives on the factors influencing the development of EPLTs for autistic children. Two dominant subthemes were identified: (1) increasing social urgency associated with escalating emotional difficulties among autistic children, and (2) broadly optimistic attitudes toward the expanding research and innovation surrounding emotion-oriented, play-based interventions.

#### 3.5.1 Urgent needs arising from escalating emotional problems

Nearly all participants expressed deep concern about the escalation of emotional difficulties among autistic children, identifying these difficulties as one of the most pressing challenges faced by this population. Coding revealed that children frequently experience sudden emotional outbursts triggered by task frustration, perceived stress, or minor changes in daily routines.

Participants described common manifestations—such as restlessness, aggression toward others, self-injurious behavior, and destructive handling of objects—as largely uncontrollable and unpredictable, thereby compounding the difficulties faced by primary caregivers and teachers.

In addition, communication differences commonly associated with autism can hinder parents' and teachers' accurate perception of children's emotional states, thereby perpetuating a cycle of unclear triggers → emotional outbursts → lack of targeted intervention. Representative examples are presented in Table 8.

**Table 8:** Increasing severity of emotional problems among autistic children.

Illustrative quotes	Participant ID
*She is also easily bored. She can't sit still for long, maybe just 15 minutes at most before she starts acting out*.	P1
*The most is letting them use my phone, usually at restaurants to keep them quiet. They just watch cartoons on YouTube. But the problem is, when they get frustrated. For example, if the video buffers, they'll throw or even bite the phone. I've had to replace phones two or three times*.	P1
*Of course, the emotional or behavioral problems of children with autism are becoming increasingly severe*.	P6

#### 3.5.2 Forward-looking trends and external support

In contrast, forward-looking trends and supportive external forces were also identified as key drivers of the development of EPLTs for autistic children. Most participants expressed positive attitudes toward this direction.

Amid increasingly severe emotional and behavioral challenges among autistic children, both parents and teachers emphasized the need to strengthen relevant intervention strategies and coping mechanisms. They anticipated that emotion-focused research and the design of EPLTs would become priority areas for future development and scholarly attention. Representative quotations are presented in Table 9.

**Table 9:** Support for forward-looking trends.

Illustrative quotes	Participant ID
*Yes, It will become mainstream because as autistic parents, we are crave for any new tech that can improve the quality of life, especially for autistic child*.	P3
*Yes, I do believe emotional counseling toys will become mainstream in the next 5 years*.	P12
*Yes, I believe emotional counseling toys have strong potential to become a mainstream trend in the next five years. As awareness about mental health and emotional needs of autistic children grows, more parents, schools, and therapists are looking for tools that go beyond just play—they want toys that can support emotional regulation, communication, and wellbeing*.	P14

### 3.6 Design factors of EPLTs for autistic children

According to the coding results, participants emphasized that the design of EPLTs for autistic children should align with the principles of the SDGs. Specifically, design considerations should prioritize:

(1) key consumer purchase decisions;
(2) the core functions and innovative features of play-learning tools;
(3) existing pain points and gaps in the play-aid market;
(4) the cost and feasibility of design and production.

#### 3.6.1 Key purchase decisions

Participants—comprising both parents of autistic children and frontline institutional teachers—represented stakeholders who collectively engage with these children in more than 80% of their daily activities. These two closely involved groups served as the primary decision-makers influencing the selection and adoption of EPLTs.

Coding analysis revealed that price, functionality, safety, and ease of use were the most critical evaluation criteria guiding consumer purchasing decisions. Parents and teachers consistently emphasized that affordability and practical utility are decisive factors determining whether such products are adopted and sustained in real-world contexts (Table 10). Representative quotations are presented in Table 10.

**Table 10:** Key purchasing decisions.

Illustrative quotes	Participant ID
*It definitely depends on whether it can help the child, then the price, and then safety*.	P28
*If others say it's quite good, I might consider buying it*.	P24
*The keywords that can entice me to make a purchase are: Safe, affordable, and child-friendly*.	P1

#### 3.6.2 Core functions and design requirements

As key decision-makers, participants identified a wide range of expected design factors for play-learning tools and related products. They emphasized the incorporation of collaborative and monitoring features that engage both parents and teachers, helping to compensate for parents' limited professional knowledge and to facilitate a triangular intervention model among parents, children, and teachers.

Participants further emphasized the importance of data-recording capabilities to document the states and frequencies of children's emotional and behavioral episodes. Such information enables parents and teachers to monitor real-time conditions and adjust intervention strategies accordingly.

Regarding design details, participants expressed expectations for a personalisable appearance, appropriate sizing, age suitability, simple operation, and interactive engagement. Price was also identified as a significant consideration: most participants expected EPLTs to be affordable for average-income families, in line with the SDGs principle of inclusiveness and accessibility.

For intended usage contexts, most participants preferred tools that could be used conveniently in small spaces, such as homes and classrooms.

Finally, with respect to innovation, participants expressed a desire for the incorporation of novel functions and new intervention mechanisms, indirectly reflecting a shared perception that the current play-aid market lacks innovation and diversity. Representative coded excerpts are presented in Table 11.

**Table 11:** Detailed design requirements for EPLTs.

Illustrative quotes	Participant ID
*It is hoped to find a new treatment method*.	P6
*Autistic children love to touch things, especially small objects. My son used to run around and touch everything, like mannequins in shops. I think he was trying to figure out if they were real humans or not. So, the toy should have different textures and sensory elements that parents can observe what the child likes to touch, how they react, and what is their gestures*.	P2
*Most importantly, I want to know where he is. How his voice or emotions sound—whether he is screaming or normal—and whether there are specific times when his emotions become unstable*.	P7

#### 3.6.3 Market constraints and pain points

Participants provided diverse feedback on the current play-aid market, revealing several persistent constraints and deficiencies. Coding analysis indicated that play-learning tools incorporating technological components were frequently excluded from consumer consideration because of their high purchase prices and ongoing maintenance costs.

Participants further criticized the limited interactivity, restricted functionality, and inadequate personalization of existing products. The absence of EPLTs in the mainstream market was regarded as a key indicator of misaligned marketing strategies and insufficient responsiveness to user needs.

In addition, participants emphasized that design-related factors—such as appearance, materials, and modes of use—can inadvertently provoke boredom, stress, or adverse emotional reactions among autistic children. The lack of unified design standards within the current market was therefore identified as a critical issue requiring immediate attention. Representative quotations are summarized in Table 12.

**Table 12:** Market pain points.

Illustrative quotes	Participant ID
*Some toys even come with candies at the bottom like those toy holders with sweets inside. My child once shoved those candy beads up his nose! It's just too risky*.	P1
*Currently the market prices are generally a bit on the high side*.	P6
*I think for the shortcoming of existing toys is almost all of them are not interactive enough. For example, if he says, “I'm angry,” the toy should help calm him down instead of doing nothing or just mimic his voices. For the existing toys, they don't understand his emotions well. There is no feedback from the toys*.	P9

#### 3.6.4 Price range and cost considerations

The vast majority of participants expressed that the price of EPLTs should align with their actual functions and production costs, with the core principle being affordability for ordinary-income families. Regarding price range expectations, participants from both China and Malaysia, based on their respective national purchasing power, formed relatively consistent expectations. They generally hope that the price of basic EPLTs would be controlled within 50–200 purchasing power units, while products with advanced features such as data recording and intelligent feedback should not exceed 300–500 purchasing power units. This range is considered reasonable and achievable for middle-income families, aligning with the principles of affordability and inclusivity advocated within the framework of the SDGs.

Participants indicated that, given the current lack of adequate government financial support, pricing must fully account for the actual economic pressures faced by families of children with autism. These families need to allocate continuous funding toward professional rehabilitation, education, and other aspects of their child's care, leaving a very limited budget for play-learning tools. If product prices are too high, even tools with excellent functionality will struggle to achieve market penetration. At the same time, participants emphasized the importance of “cost-effectiveness,” stating that product pricing must balance quality and functionality while rejecting “premium pricing” for unnecessary design features. Some participants expressed the view that “you get what you pay for,” acknowledging that reasonable price increases corresponding to verifiable improvements in functionality are acceptable, provided that the product's effectiveness can be demonstrated and that usage costs remain low (no high maintenance fees or consumable expenses). Additionally, some Malaysian participants expressed hope that the government or social organizations would introduce policies such as price subsidies or public welfare distribution programs to further reduce families' acquisition costs, enabling more children with autism to benefit from the support of EPLTs. Representative interview excerpts are presented in Table 13.

**Table 13:** Price positioning.

Illustrative quotes	Participant ID
*You get what you pay for; prices ranging from a few dozen to 200–300 are acceptable*.	P5
*I think a fair price would be 50–200 for basic versions, and up to 300–500 for advanced versions with tech—but only if they're truly effective*.	P12
*As long as it's not too expensive, and “too” means exceeding the family's financial capacity here at home, we don't want it to be too high*.	P24

### 3.7 Attitudes toward the integration of advanced technologies in EPLTs

Coding comparison revealed that participants held ambivalent attitudes toward the integration of advanced technologies into play-learning tools. On the one hand, some participants believed that technological incorporation could broaden functionality and enhance personalization and appeal. On the other hand, others expressed concern that technological dependence might negatively influence children's social interactions with real people and reduce opportunities for natural engagement.

#### 3.7.1 Support for technological integration

Approximately half of the participants expressed positive attitudes toward incorporating advanced technologies—such as virtual reality (VR), augmented reality (AR), and artificial intelligence (AI)—into play-learning tools. They believed that embedding technological features could expand interactive engagement and enhance personalization, enabling these tools to deliver customized feedback that accommodates individual differences among autistic children.

Several participants also noted that such technological elements could strengthen connections between parents and children, bridging communication gaps and promoting more interactive engagement. Representative quotations are presented in Table 14.

**Table 14:** Support for technological integration.

Illustrative quotes	Participant ID
*I introduced my son to an iPad, and now he operates it even better than me. He changes settings I don't even know how to adjust. Even though I only use my phone for basic apps like WhatsApp, he can manage more. He even plays mobile games. So, I believe children, even with autism, can benefit a lot from modern tools*.	P2
*There's nothing wrong with trying this AI toy—I strongly support it. There are not many play-based interventions available for autism*.	P7
*I strongly agree. For example, VR can take him to places we cannot afford to bring him, such as the zoo or Legoland. If books are equipped with AR, it can help him understand the objects or stories in the book more effectively*.	P8

#### 3.7.2 Concerns about incorporating advanced technologies

Some participants expressed reservations about incorporating advanced technological elements into play-learning tools and related products. They cautioned that introducing electronic and information technology components could diminish children's social communication abilities and increase levels of fatigue and stress.

Several respondents also expressed concern that exposure to technologically enhanced play tools might foster dependence on such devices, reducing opportunities for interaction with real people and potentially producing counterproductive effects on therapeutic interventions.

In addition, participants acknowledged that the use of advanced technologies inevitably generates data, raising privacy concerns—identified by many parents as among the most sensitive and worrisome issues. Representative quotations are presented in Table 15.

**Table 15:** Opposition to technological integration.

Illustrative quotes	Participant ID
*When you're developing something a child can engage with, it should give parents some comfort too that the child has something else to rely on, not just humans*.	P2
*Previously, my child was very addicted to gadgets. So I stopped him from playing with them, and it has now been six months. Alhamdulillah, many positive changes have taken place*.	P7
*But my main concern is still data privacy. Therapists should be involved, since we cannot rely only on technology. But how can we make sure the privacy is important. We don't want to share all data*.	P3
*My concern mainly on its durability and whether the cost would be affordable*.	P11

### 3.8 Comparative analysis of coding frequencies across five primary themes between China and Malaysia

**Figure 1:**
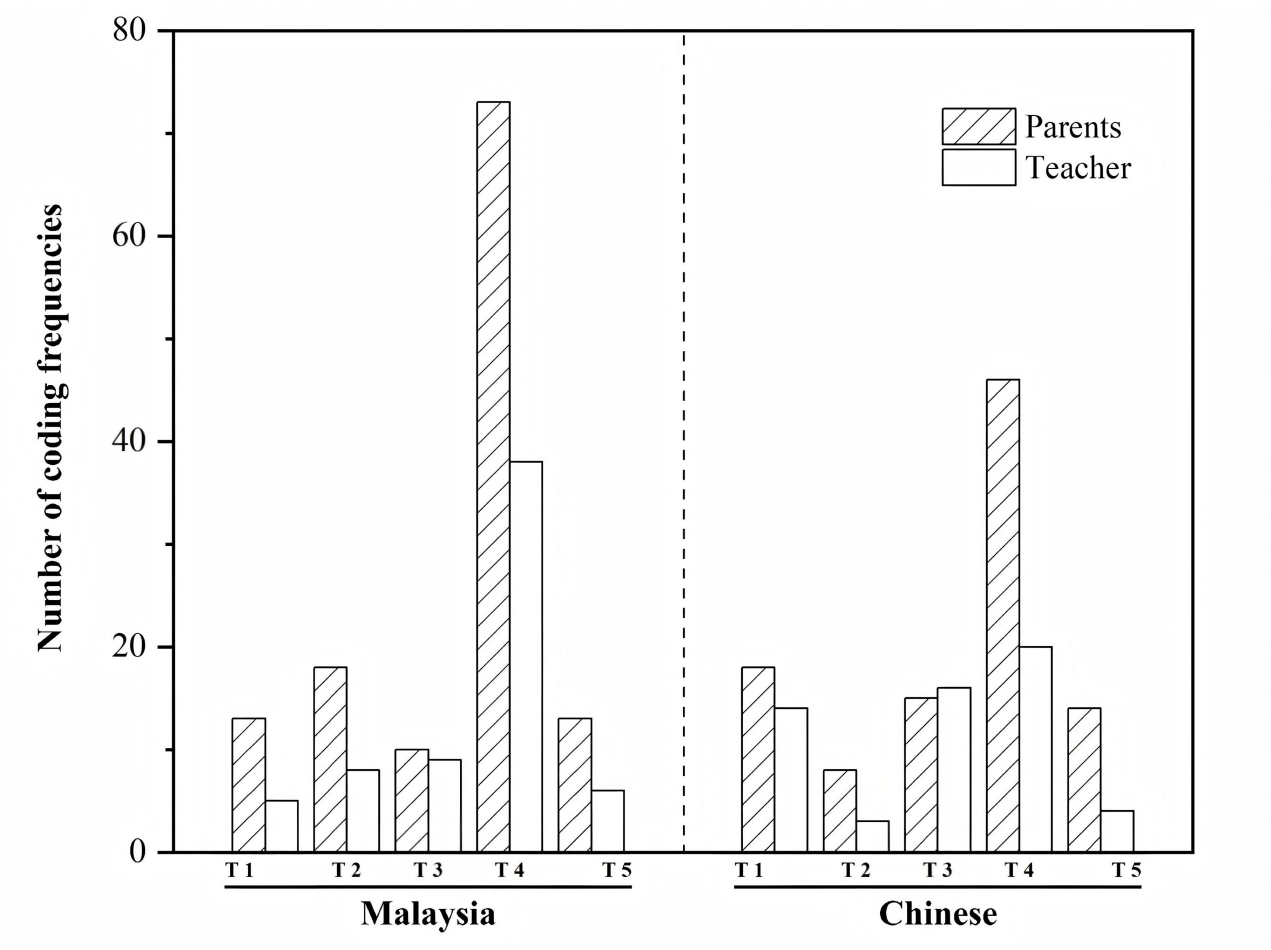
Comparative analysis of coding frequencies across five primary themes between China and Malaysia.

Drawing on NVivo frequency statistics presented in Table 16 and Figure 1—which compare the occurrence rates of the five primary themes across China and Malaysia—clear variations emerged by country and respondent role (parents vs. teachers). These differences reflect not only the sociocultural and institutional contexts of the two nations but also the distinct experiential logics through which parents and teachers engage with EPLTs.

**Table 16:** Coding frequency statistics of participants.

	Malaysian parents	Malaysian teachers	Chinese parents	Chinese teachers
T1	13	5	18	14
T2	18	8	8	3
T3	10	9	15	16
T4	73	38	46	20
T5	13	6	14	4

Data were derived from NVivo-coded interview transcripts with parents and teachers from China and Malaysia (T = Theme). T1, Factors driving the design and development of EPLTs for autistic children; T2, Consumer attitudes toward the integration of advanced technologies; T3, Potential and applications of EPLTs; T4, Design and market requirements for EPLTs; T5, Socio-environmental issues affecting autistic children.

Overall, the Chinese sample demonstrated consistently higher coding frequencies across all five themes, indicating stronger problem awareness and greater expressive density among Chinese respondents. In contrast, the Malaysian sample displayed a more dispersed code distribution, suggesting a preference for practical experience and concrete case descriptions rather than abstract or policy-oriented reflections.

(1) **Theme 1: Factors driving the design and development of EPLTs for autistic children**

Chinese respondents generated 32 references, compared with 18 from Malaysian participants. Within China, contributions from parents (31 codes) and teachers (19 codes) were both substantial. This pattern suggests that participants in both countries recognized social trends and the intensification of children's emotional difficulties as key drivers of play-aid development, albeit with different emphases.

Interview narratives indicated that Chinese respondents tended to foreground structural and institutional factors—such as insufficient governmental support, policy delays, and heightened family stress resulting from escalating emotional challenges. In contrast, Malaysian respondents adopted a more grassroots, community-based perspective, describing development as emerging from parental collaboration, community self-help, and local innovation initiatives.

This divergence highlights differences in the maturity of institutionalized support systems: China's higher thematic frequency signals systemic resource pressure, whereas Malaysia's lower frequency reflects a flexible and informal compensation mechanism embedded within community networks.

(2) **Theme 2: Consumer attitudes toward advanced technology integration**

This theme revealed a marked contrast between China and Malaysia. Malaysian participants generated 26 codes, substantially exceeding the 11 contributed by Chinese respondents. Malaysian parents and teachers expressed greater openness to integrating advanced technologies—such as VR, AR, and AI—into EPLTs, believing that such tools could enhance personalization, playfulness, and educational utility, thereby improving children's learning engagement and emotional participation.

In contrast, Chinese respondents adopted a more cautious stance, citing concerns about data privacy, technological dependence, and the potential substitution of human interaction. Some parents feared that over-technologisation might reduce children's natural social interactions or lead to excessive attachment and overuse.

This contrast highlights fundamental differences in cultural and institutional trust structures. Malaysia's pluralistic education system and relatively low levels of digital anxiety promote a pragmatic and instrumental acceptance of technological tools, whereas the heightened vigilance among Chinese respondents reflects a strong sensitivity to issues surrounding technology, ethics, and child safety. Accordingly, explainability and user control emerged as decisive conditions for the acceptance of advanced technologies in both educational and therapeutic contexts.

(3) **Theme 3: Potential and applications of EPLTs**

For this theme, the difference between China (31 references) and Malaysia (19 references) was moderate, yet the two groups diverged semantically. Chinese teachers emphasized institutional and classroom applications, noting the value of these tools for identifying children's emotional states and regulating classroom pacing. In contrast, Malaysian parents focused on family-based emotional companionship, highlighting their roles in soothing, comforting, and facilitating emotional expression.

At a broader sociocultural level, the Chinese discussion aligned with a logic of institutionalized intervention, positioning these tools within structured systems of professional and early therapeutic support. Meanwhile, the Malaysian discussion reflected a logic of daily support, framing the tools as tools for emotional communication and parent–child bonding. This divergence underscores the need to balance formal intervention with domestic practice in future design and dissemination avoiding over-institutionalization on the one hand and over-domestication on the other.

(4) **Theme 4: Design and market requirements for EPLTs**

This theme appeared most frequently across the dataset, highlighting shared concerns among parents and teachers regarding affordability, functionality, and design details. Across both countries, the combined frequency reached 111 references, with the Chinese sample contributing fewer codes than the Malaysian sample. Parents (119 references) outnumbered teachers (58); given the 2:1 participant ratio (20 parents vs. 10 teachers), both groups demonstrated broadly comparable engagement with this topic.

Chinese parents primarily emphasized price, functionality, and safety standards, reflecting financial strain and limited policy support. In contrast, Malaysian participants prioritized cultural adaptability and personalized design, valuing tools that reflected children's interests and promoted social inclusiveness.

Across both contexts, a common market challenge emerged: weak standardization, high costs, and maintenance difficulties that hinder sustainable use. Consumer priorities have shifted from “whether products exist” to “whether they are usable, trustworthy, and sustainable,” signaling a growing demand for quality assurance and evidence-informed design aligned with global trends in inclusive-education product development.

Implications: Future design and dissemination should prioritize baseline standards—safety, maintainability, and affordability—and include summaries of real-world evidence (RWE) alongside product claims to strengthen trust and support sustainable adoption.

(5) **Theme 5: Socio-environmental issues affecting autistic children**

Coding frequencies for socio-environmental issues were similar across the two countries (China: 18; Malaysia: 19), but the interpretive focus diverged. Teachers in both contexts more frequently discussed inefficient government policies and unequal resource allocation, reflecting how institutional constraints shape teaching and intervention practices. In contrast, parents more often raised concerns about public perceptions and social stigma, citing limited social acceptance and a lack of autism-friendly public spaces.

These differences indicate that socio-environmental barriers manifest at distinct levels: in China, challenges are predominantly institutional and policy-related, whereas in Malaysia they are more closely linked to cultural acceptance and public awareness. Improving the social environments of autistic children therefore requires both structural reform and public education—combining policy responsiveness with sustained efforts to raise awareness and reduce stigma through inclusive community initiatives.

Implications: Pair upstream policy and resource reforms with community-level stigma-reduction campaigns and autism-friendly space design to achieve measurable improvements in participation.

## 4 Discussion

### 4.1 Socio-environmental challenges of autistic children: reframing from “individual difficulties” to “systemic conditions”

Existing research on the causes of emotional problems in children with autism has often fallen into the misconception of attributing them solely to “individual child differences.” For instance, (Hor et al. 2025) primarily focused on the relationship between emotional outbursts and children's own cognitive deficits and behavioral disorders, while neglecting the systemic influence of the external environment. In contrast, the social constructivist theory of disability posits that disability is not an individual deficit but rather the result of the interaction between social environments, institutional arrangements, and individual characteristics (Oliver, 2013). The narratives from respondents in both China and Malaysia in this study—regarding discrimination against children with autism, fragmentation of resources, and inefficient government support—are highly consistent with this theoretical perspective. They further reveal that the emotional challenges faced by children with autism are not purely a matter of “individual child factors,” but are instead triggered by systemic factors in the external environment. This systemic predicament also directly constrains the promotion and application of EPLTs.

When governmental and institutional responses remain confined to “post-outburst noise reduction” or “teacher-dependent control,” structural stress is effectively transferred to children and their families. This dynamic perpetuates an institutional–government feedback loop—behavioral outburst → reactive intervention → ineffective feedback—and further reinforces the moralized narrative that parents or teachers are “insufficiently professional.” This overlooks the core responsibility of schools, public spaces, and grassroots support systems in mitigating the sources of emotional triggers at the front end. Research by (Doda et al. 2024) also confirms that emotional problems in children with autism are often related to the absence of social support systems and the unfriendliness of public environments. This finding corroborates the experiences reported by respondents in this study, such as “children being easily triggered when going out” and “lack of family resources.”

From a critical standpoint, this approach is both unjust and inefficient: it obscures the accountability of school organizations, public spaces, and grassroots service systems in addressing the antecedent causes of emotional overload, and it undermines the systemic capacity for preventive relief and structural buffering.

Social discrimination and the unfriendliness of public spaces make children with autism highly susceptible to emotional outbursts triggered by environmental stimuli when they go out. Meanwhile, families, burdened by financial pressures and a lack of resources, are unable to obtain suitable portable EPLTs, which further exacerbates the children's emotional challenges. This aligns with the findings of (Anderson et al. 2025) and (Bhuiyan and Islam 2025) in their research on families of children with autism, namely that economic burden and insufficient resource availability are key factors constraining the effectiveness of family interventions. The issue raised by Malaysian respondents regarding “insufficient resources and a shortage of teaching staff in public institutions” is also consistent with recent studies (Diaz and Madrigal, 2025; Sarwat and Biswal, 2025). Currently, autism support services generally face the dual challenge of teacher shortages and an inadequate supply of play-learning tools. This prevents teachers from providing personalized emotional support to children and hinders the formation of collaborative home-school intervention models. The lack of government policy, absent standards, and insufficient subsidies not only lead to variable quality of EPLTs on the market but also make it difficult for ordinary families to afford high-quality products. This creates a market contradiction characterized by “urgent demand yet insufficient supply.”

Therefore, the source-level governance of the social environment for children with autism should be proactively implemented at the level of daily public affairs. This perspective extends Oliver's (2013) social constructivist theory of disability by translating environmental optimization from “macro-level advocacy” to “specific contexts”: promoting the development of autism-friendly spaces (such as quiet corners and transition areas in public settings) to reduce emotional triggers caused by environmental stimuli. At the school level, transition periods, class sizes, and classroom pacing should be built into daily scheduling processes, with adequate provision of EPLTs and professional training for teachers on their use, thereby enhancing the effectiveness of institutional interventions. This aligns with the “school-family-community collaborative intervention” concept discussed in the research by (Neil and Villani 2025).

At the policy level, local governments should establish clear design and usage standards for play-based tools and assistive products for autistic children. These standards should be supported by financial subsidies, parent–teacher training programmes, and coordinated funding mechanisms to mitigate inefficiencies arising from weak institutional feedback loops. Only by shifting from end-stage remediation to front-end systemic regulation and relief can actors involved in autism intervention—including institutional teachers, parents, and play-learning tools—operate within realistic and clearly defined boundaries, contributing stable, interpretable, and complementary roles within the broader support ecosystem.

### 4.2 Driving factors: “serious emotional problems” as the catalyst for trend expectations

Across all interview codes, participants consistently emphasized that emotional difficulties among autistic children have become increasingly serious—characterized by high frequency, high intensity, and pronounced contextual dependency and unpredictability. These episodes typically arise from two intertwined dimensions: the child's own behavioral and physiological thresholds (e.g., sudden outbursts, unpredictable moods, and restlessness) and high-load environments (e.g., fatigue, perceived pressure, and sensory overload). Consistent with prior research, its characteristics also include factors such as low trigger thresholds and prolonged recovery periods following calming (Northrup et al., 2020). This study further contributes cross-cultural empirical evidence from both China and Malaysia. In other words, the interplay between an individual's fragile physiological and behavioral thresholds and the high-stimulus external environment constitutes an emotional–behavioral system characterized by a “low trigger threshold—rapid escalation—slow recovery” pattern.

Consequently, both parents and teachers viewed emotional dysregulation as the most pressing challenge, driving innovation and demand for emotion-oriented, play-based tools and related intervention products. This echoes the research findings of (Wang and Jeon 2024), which suggest that the urgency and authenticity of demand on the user side are core drivers of innovation in assistive devices for autism. It is noteworthy—and warrants caution—that this study simultaneously found: from a market perspective, the urgency parents feel to address their children's emotional problems can easily lead to impulse purchases and a technology worship regarding EPLTs for children with autism. Existing research has largely focused on “product function innovation” while overlooking the irrational factors behind purchasing behavior and the need for real-world evidence (Wang and Jeon, 2024; Zhou et al., 2025). This oversight has normalized a negative cycle of “stress → purchase → sunk costs → forced continued use.” This dynamic reflects an affective form of consumerism in which intervention decisions are shaped more by anxiety and trend perception than by empirical evidence or sustained usability. This suggests that subsequent research should focus on the balance between “urgency of demand and rational purchase,” providing a new perspective for product promotion and market regulation.

### 4.3 Design requirements centered on “safety, affordability, recordable collaboration, and personalization”

Analysis of participant feedback and market pain points indicates that many purported “innovations” are introduced without adequate consideration of maintenance burdens, consumables, or user training. The principal barriers to sustainable adoption are therefore less about functionality and more about insufficient evidence of effectiveness, limited affordability, and poor maintainability (Fernández-Batanero et al., 2022; Field and Jette, 2007).

At a structural level, emotion-oriented, play-based tools for autistic children remain under-represented in the market, reflecting both the low perceived return on investment and the systemic resource deprivation affecting this population. Importantly, this phenomenon aligns with the global situation outlined in the “Global report on assistive technology” by the World Health Organization and United Nations Children's Fund (2022), which highlights the insufficient supply of assistive devices for autism and the uneven distribution of access to assistive technology resources among families and teachers. This reflects the difficult situation faced by children with autism due to the lack of resources in both the market and policy frameworks.

Accordingly, EPLTs innovation should be guided by a “triangle constraint” of evidence–affordability–quality: any new function must be stress-tested against the weakest side of the triangle so that costs, safety, and training requirements are realistically integrated into decision-making. A tiered promotion model—trial → purchase → iteration—would enable schools and families to test products in small groups to determine contextual fit and effectiveness. This perspective expands the application boundaries of the user-centered design (UCD) concept (Heimgärtner, 2020) and helps to establish a real-world evidence (RWE) feedback loop, thereby preventing “imagined design” mismatches between product features and real-world contexts.

In parallel, we suggest promotion community resource-sharing mechanisms—such as institutional lending schemes, local tool libraries, and second-hand circulation—could mitigate resource inequalities and enhance adoption equity, transforming “urgent demand” into “verifiable and sustainable innovation.” This responds to the call for “strengthening the accumulation of evidence on research effectiveness” (Al-Hendawi et al., 2023; Parsons and Cobb, 2011; Scherer and Federici, 2015). Viewed longitudinally, surface-level concerns about price, functionality, and safety ultimately highlight the need for a baseline design standard for autism-related products that are *usable in daily life*. This standard should include: Safety: non-toxic materials, controlled edge profiles, and minimal choking risk; Sensory interactivity: calibrated visual, tactile, vibratory, and auditory feedback, together with clear emotional-support cues; Low burden/low stress: minimal noise and odor, discreet appearance to reduce stigma, portability, ease of storage, and cost control.

At the same time, users continue to expect innovation. The most frequently mentioned design expectations were: Collaborative features that enable parents and teachers to share minimal essential cues or event-level data templates, reducing communication friction; Data-recording functions that capture interaction metrics such as contact frequency, emotional thresholds, duration, and stress indicators.

With regard to appearance and usage contexts, participants preferred play-learning tools featuring personalisable aesthetics and proportional sizing, allowing adaptation to individual differences and sustaining baseline emotional engagement through familiarity and appeal.

### 4.4 Attitudes toward advanced technological elements: balancing “explainability, controllability, and switchability”

Participants' attitudes toward integrating advanced technological elements—such as VR, AR, and AI—were shaped by three dimensions of uncertainty: explainability, controllability, and switchability. Among these, “explainability” refers to the transparency of the algorithmic recommendation logic, “controllability” means that parents/teachers can adjust the intensity of the technological features, and “switchability” indicates the ability to switch with one click to a basic mode without technology. This aligns with the core perspectives of ethical AI research; studies by (Cheung and Ho 2025) and (Xu and Wang 2024) both mention that the explainability and controllability of AI technology can earn user trust. When recommendation logic is opaque, data flows are unclear, or operating modes cannot be easily turned off, parents and teachers expressed concern about being displaced by embedded algorithmic mechanisms. Such uncertainty fosters a pervasive sense of lost control and mistrust in the information and feedback generated by these devices.

For example, participants described scenarios in which a child might use a technologically enhanced play-based teaching aid with speech-interaction capabilities in the absence of parental supervision. If the device were to deliver inappropriate or misleading prompts, the child's safety could be compromised. Consistent with some studies, participants also raised privacy and data-security concerns, particularly regarding the potential leakage or misuse of recorded information (Liu et al., 2025).

Coding further revealed concerns—voiced by a minority—about cost and affordability, questioning whether products equipped with advanced features would remain accessible to average-income families. From a sustainability perspective, participants emphasized that pricing should align with Sustainable Development Goal (SDG) 10: Reduced Inequalities (Ebbesson and Hey, 2022), ensuring that assistive technologies for autistic childrenremain equitable and affordable across diverse socioeconomic contexts.

On the other hand, participants also acknowledged several potential benefits. Advanced technologies could compensate for parents' limited professional expertise and serve as a bridge for communication between parents and children, thereby supporting the envisioned parent–child–teacher triangular collaboration model within intervention settings (Raudeliunaite and GudŽinskiene, 2023). In addition, technological integration was perceived to enhance functionality, personalization, and overall appeal, making EPLTs more engaging for children while simultaneously stimulating consumer interest among parents and teachers. This dual effect may increase public visibility and attract investment attention to the assistive-technology market for autistic children. This further confirms the role of technology in enhancing product appeal and market vitality (Zayer et al., 2025).

The differences in attitudes toward high-tech between China and Malaysia essentially reflect the broader differences in the overall models of emotional support systems for autism in the two countries. It is worth noting that the differences in technology acceptance between China and Malaysia are not absolute opposites but rather represent a “degree of difference” rather than a “fundamental disagreement.” The cautious attitude of Chinese respondents does not signify a rejection of technology, but rather places higher demands on the “boundaries” of technology—it must be clearly positioned as an auxiliary tool in interventions, and cannot replace interpersonal interaction and professional guidance. The open attitude of Malaysian respondents is also based on “functional practicality” and does not represent an indiscriminate pursuit of all high-tech elements. This consensus suggests that the core principle of integrating technology should be “empowerment rather than replacement.” This finding of cross-cultural differences preliminarily addresses the limitations of existing research: previous studies on technology acceptance in autism have mostly focused on Western high-income countries or single countries (de Leeuw et al., 2020). Through a comparison between China and Malaysia, this study reveals the distinct characteristics of technology acceptance under different social support systems. It also echoes the theoretical perspective in cross-cultural psychology that “cultural background influences technology acceptance” (Masimba et al., 2019), providing empirical support for the subsequent design and promotion of cross-cultural assistive products for autism.

## 5 Limitations

This study adopted a cross-cultural, multi-site qualitative design with a relatively small sample size. Although thematic saturation was achieved, the findings are not intended to be generalized to the population level. The use of online interviews may have limited access to non-verbal cues, and translation between Chinese, English, and Malay may have introduced potential semantic bias. Furthermore, the researchers' disciplinary backgrounds may have influenced both the framing of interview questions and the interpretation of participants' narratives.

To mitigate these limitations, dual independent coding, peer debriefing, and negative case analysis were employed, and representative quotations were provided to enhance interpretive transparency and credibility.

## 6 Conclusion

In summary, NVivo coding revealed higher overall frequencies among Chinese participants, indicating heightened attention and greater expressive density under conditions of policy transformation and social pressure. Although the Malaysian sample demonstrated lower frequencies, it displayed a more distinctive practice orientation, emphasizing family-based action and teacher-led innovation.

By role, parents' voices were most prominent within the themes of design requirements and driving factors. This finding suggests that parents are not only primary consumers but also emerging co-creators of design knowledge for EPLTs. A critical implication follows: the emotional support system for autistic children cannot rely solely on policy or institutional interventions; it must also acknowledge micro-level practices embedded within family and cultural contexts. When research and market initiatives lean too heavily on institutionalized or standardized intervention logics, they risk overlooking lived family experiences and the social–emotional networks that sustain development.

Overall, the Chinese sample reflected a strong desire for institutionalization and functional optimization, mirroring structural resource constraints and gaps between social expectations and practical realities. By contrast, the Malaysian sample's lower frequency reflected a family–community-driven model characterized by flexible and context-sensitive support strategies developed under limited institutional backing.

These two modes—top-down, policy-driven and bottom-up, practice-driven—represent complementary pathways for understanding and enhancing emotional support. Future research and product development should integrate both approaches, advancing cross-cultural, human-centered, and empathetic design frameworks that balance functional efficacy with emotional support. Such integration would facilitate the transition of EPLTs from mere availability to evidence-informed effectiveness, supported by real-world evidence (RWE) cycles and user-centered implementation.

## Figures and Tables

**Table A1:** Standards for reporting qualitative research (SRQR) checklist.

Title and abstract	Coverage
**Title**—Concise description of the nature and topic of the study Identifying the study as qualitative or indicating the approach (e.g., ethnography, grounded theory) or data collection methods (e.g., interview, focus group) is recommended	Yes
**Abstract**—Summary of key elements of the study using the abstract format of the intended publication; typically includes background, purpose, methods, results, and conclusions	Yes
Introduction	
**Problem formulation**—Description and significance of the problem/phenomenon studied; review of relevant theory and empirical work; problem statement	Yes
**Purpose or research question**—Purpose of the study and specific objectives or questions	Yes
Methods	
**Qualitative approach and research paradigm**—Qualitative approach (e.g., ethnography, grounded theory, case study, phenomenology, narrative research) and guiding theory if appropriate; identifying the research paradigm (e.g., postpositivist, constructivist/ interpretivist) is also recommended; rationale**	Yes
**Researcher characteristics and reflexivity**—Researchers' characteristics that may influence the research, including personal attributes, qualifications/experience, relationship with participants, assumptions, and/or presuppositions; potential or actual interaction between researchers' characteristics and the research questions, approach, methods, results, and/or transferability	Yes
**Context**—Setting/site and salient contextual factors; rationale	Yes
**Sampling strategy**—How and why research participants, documents, or events were selected; criteria for deciding when no further sampling was necessary (e.g., sampling saturation); rationale**	Yes
**Ethical issues pertaining to human subjects**—Documentation of approval by an appropriate ethics review board and participant consent, or explanation for lack thereof; other confidentiality and data security issues	Yes
**Data collection methods**—Types of data collected; details of data collection procedures including (as appropriate) start and stop dates of data collection and analysis, iterative process, triangulation of sources/methods, and modification of procedures in response to evolving study findings; rationale**	Yes
**Data collection instruments and technologies**—Description of instruments (e.g., interview guides, questionnaires) and devices (e.g., audio recorders) used for data collection; if/how the instrument(s) changed over the course of the study	Yes
**Units of study**—Number and relevant characteristics of participants, documents, or events included in the study; level of participation (could be reported in results)	Yes
**Data processing**—Methods for processing data prior to and during analysis, including transcription, data entry, data management and security, verification of data integrity, data coding, and anonymization/de-identification of excerpts	Yes
**Data analysis**—Process by which inferences, themes, etc., were identified and developed, including the researchers involved in data analysis; usually references a specific paradigm or approach; rationale**	Yes
15.5-7.4,-13.5175.3mm**Techniques to enhance trustworthiness**—Techniques to enhance trustworthiness and credibility of data analysis (e.g., member checking, audit trail, triangulation); rationale	Yes
Results/findings	
**Synthesis and interpretation**—Main findings (e.g., interpretations, inferences, and themes); might include development of a theory or model, or integration with prior research or theory	Yes
**Links to empirical data—**Evidence (e.g., quotes, field notes, text excerpts, photographs) to substantiate analytic findings	Yes
Discussion	
**Integration with prior work, implications, transferability, and contribution(s) to the field—**Short summary of main findings; explanation of how findings and conclusions connect to, support, elaborate on, or challenge conclusions of earlier scholarship; discussion of scope of application/generalizability; identification of unique contribution(s) to scholarship in a discipline or field	Yes
**Limitations**—Trustworthiness and limitations of findings	Yes
Other	
**Conflicts of interest**—Potential sources of influence or perceived influence on study conduct and conclusions; how these were managed	Yes
**Funding**—Sources of funding and other support; role of funders in data collection, interpretation, and reporting	Yes
The authors created the SRQR by searching the literature to identify guidelines, reporting standards, and critical appraisal criteria for qualitative research; reviewing the reference lists of retrieved sources; and contacting experts to gain feedback. The SRQR aims to improve the transparency of all aspects of qualitative research by providing clear standards for reporting qualitative research.	Yes
15.5-7.4,-27175.3mmThe rationale should briefly discuss the justification for choosing that theory, approach, method, or technique rather than other options available, the assumptions and limitations implicit in those choices, and how those choices influence study conclusions and transferability. As appropriate, the rationale for several items might be discussed together.	Yes
Reference	
O'Brien BC, Harris IB, Beckman TJ, Reed DA, Cook DA. Standards for reporting qualitative research: a synthesis of recommendations. Academic Medicine, Vol. 89, No. 9 / Sept 2014 doi: 10.1097/ACM.0000000000000388	Yes

## Data Availability

The original contributions presented in the study are included in the article/Supplementary material, further inquiries can be directed to the corresponding author.
