# Supplementary material for: Design and application of emotion-oriented play-learning tools for children with autism: a cross-cultural qualitative study of parents and teachers in China and Malaysia
Source: Front Psychol. 2026 Apr 30;17:1736058. doi: 10.3389/fpsyg.2026.1736058 (PMC13171507; doi:10.3389/fpsyg.2026.1736058)
Supplement: Supplementary file 1 [file Table_1.docx]

# **Supplementary Material**. Recruitment Flowchart

Start

↓

Identification of recruitment sites

- Special schools (China, Malaysia)

- ASD support centers

- Parent associations

- Teacher networks

↓

Dissemination of study invitation

- Posters

- WeChat/WhatsApp groups

- Institutional notice boards

- Offline visit publicity

↓

Initial screening by inclusion criteria

| 1. Parents of children diagnosed with ASD (DSM-5 Level 1) |
| --- |
| 1. Special education teachers with ≥1 year of experience |
| 1. Aged 20–55; able to communicate in Mandarin / English |
| 1. Voluntary consent |

↓

Verification of ASD diagnosis

(Confirmation by collaborating institutions)

↓

Eligibility confirmed?

↙ ↘

YES NO

↓ ↓

Scheduling of interview Excluded

↓

Completion of 30 interviews

(China: 15; Malaysia: 15)

(Parents: 15; Teachers: 15)

↓

Data saturation reached (28–30 interviews)

↓

End

**Participant Recruitment**

Maximum-variation sampling was adopted to recruit two key stakeholder groups: (a) parents/primary caregivers of children with autism spectrum disorder (ASD) and (b) education practitioners (frontline teachers working in institutions providing support for autistic children). Recruitment was conducted across designated sites in China and Malaysia, including special schools, ASD support centers, parent associations, and teacher networks. Study invitations were disseminated through multiple channels: physical posters, institutional notice boards, online groups (WeChat/WhatsApp), and offline on-site publicity activities to maximize outreach to potential eligible participants.

Following the dissemination of study information, an initial screening against predefined inclusion criteria was conducted for all interested individuals. For potential participants who passed the initial screening, the ASD diagnosis of the involved child was further verified and confirmed by collaborating professional institutions to ensure diagnostic validity. Only participants with confirmed eligibility proceeded to interview scheduling, while those failing to meet the criteria were excluded from the study. A total of 30 semi-structured interviews were completed (15 in China and 15 in Malaysia), with equal representation of the two stakeholder groups (15 parents and 15 teachers). Data collection was terminated when data saturation was achieved (between 28 and 30 interviews), indicating no new thematic insights could be derived from additional participants.

**Eligibility Criteria**

**Inclusion Criteria**

1. For the parent group: Biological parents (first-degree relatives) of a child diagnosed with DSM-5 Level 1 ASD, with the child currently residing in the family; for the teacher group: Special education frontline teachers with at least 1 year of professional experience working directly with autistic children in relevant support institutions (e.g., rehabilitation centers, supportive therapy centers, early intervention centres, special schools).
2. Aged 20–55 years old, with adequate communication ability in either Mandarin or English to complete the interview.
3. Voluntarily provided informed consent to participate in the study.

**Exclusion Criteria**

1. Failure to meet the above core inclusion criteria at the initial screening stage.
2. Inability to provide official and confirmed DSM-5 Level 1 ASD diagnostic documentation for the child (parent group) or lack of verifiable 1+ year of relevant professional experience (teacher group).
3. Inability to communicate adequately in Mandarin or English, or refusal to provide voluntary informed consent for study participation.
